# The RhoA-ROCK pathway in the regulation of T and B cell responses

**DOI:** 10.12688/f1000research.7522.1

**Published:** 2016-09-12

**Authors:** Edd Ricker, Luvana Chowdhury, Woelsung Yi, Alessandra B. Pernis

**Affiliations:** 1 Autoimmunity and Inflammation Program, Hospital for Special Surgery, New York, New York, 10021, USA; 2 Graduate Program in Immunology and Microbial Pathogenesis, Weill Cornell Graduate School of Medical Sciences, New York, New York, 10065, USA; 3 David Z. Rosensweig Genomics Research Center, Hospital for Special Surgery, New York, New York, 10021, USA; 4 Department of Medicine, Weill Cornell Medical College, New York, New York, 10021, USA

**Keywords:** Autoimmune, RhoA, ROCK, Rho kinase, RhoA-ROCK axis, lymphocyte development, lymphocyte activation

## Abstract

Effective immune responses require the precise regulation of dynamic interactions between hematopoietic and non-hematopoietic cells. The Rho subfamily of GTPases, which includes RhoA, is rapidly activated downstream of a diverse array of biochemical and biomechanical signals, and is emerging as an important mediator of this cross-talk. Key downstream effectors of RhoA are the Rho kinases, or ROCKs. The ROCKs are two serine-threonine kinases that can act as global coordinators of a tissue’s response to stress and injury because of their ability to regulate a wide range of biological processes. Although the RhoA-ROCK pathway has been extensively investigated in the non-hematopoietic compartment, its role in the immune system is just now becoming appreciated. In this commentary, we provide a brief overview of recent findings that highlight the contribution of this pathway to lymphocyte development and activation, and the impact that dysregulation in the activation of RhoA and/or the ROCKs may exert on a growing list of autoimmune and lymphoproliferative disorders.

## Introduction

Effective immune responses require an intricate and dynamic cross-talk between hematopoietic and non-hematopoietic cells. Precise regulation of these interactions is necessary to efficiently clear pathogens while preventing the emergence of autoimmunity. Rho-GTPases, such as RhoA, are emerging as important mediators of this cross-talk owing to their ability to be rapidly activated downstream of a broad range of biochemical and biomechanical signals ^1, 2^. Upon activation, RhoA interacts with a number of different effector molecules, including the Rho kinases (or ROCKs), two highly homologous serine–threonine kinases that coordinate a tissue’s response to stress and injury via effects on a wide array of biological processes ^1, 3– 5^. The RhoA-ROCK pathway has, indeed, been implicated in the control of cytoskeletal reorganization and migration, proliferation, survival, and gene expression ^1, 3– 5^. Despite the fundamental reliance of T and B cells on these processes, the precise involvement of the RhoA-ROCK pathway in lymphocyte biology has not been fully elucidated. In this commentary, after briefly discussing recent work on the role of RhoA and the ROCKs in the development and activation of lymphocytes, we will highlight new findings that may link dysregulation of this pathway to a growing list of autoimmune and lymphoproliferative disorders. The reader should note that despite the well-established connection between the activation of RhoA and that of the ROCKs, their role in immune physiology and pathophysiology has often been investigated separately and thus most of the studies that will be discussed primarily focus on one or the other component of this signaling cascade.

## Regulation of the RhoA–ROCK pathway

Rho GTPases, which include the RhoA subfamily, are ubiquitously expressed molecular switches that cycle between an inactive (GDP-bound) and an active (GTP-bound) state, a process regulated by the local balance of guanine nucleotide exchange factors (GEFs, which promote the exchange of GDP for GTP), GAPs (GTPase activating proteins, which enhance the intrinsic GTPase activity), and guanosine nucleotide dissociation inhibitors (GDIs, which bind and sequester inactive GTPases in the cytoplasm) ^2^. GEF activation in response to stimuli such as chemokines, growth factors, cell–matrix interactions, and mechanical signals leads to the activation of RhoA, which can then interact with several downstream effector molecules ^1^. Over 79 GEFs have been identified in the mammalian genome, with at least 24 of them being reported to activate RhoA ^6^. This redundancy in RhoA activation allows multiple upstream signals to converge onto RhoA and may help compartmentalize specific GEFs with selected RhoA substrates into unique complexes, thus facilitating the transmission of distinct downstream effector functions ^7^.

Critical downstream effectors of RhoA are the ROCKS, ROCK1 and ROCK2, two serine-threonine kinases encoded by separate genes ^1, 3– 5^. The catalytic kinase domain is located in the N-terminus and is followed by a coiled-coil region containing the Rho-binding domain (RBD) and a pleckstrin homology domain. The C-terminus of the ROCKs interacts with the N-terminus and has autoinhibitory activity ^1, 3– 5^. Binding of activated RhoA to the RBD disrupts the association of the autoinhibitory C-terminus with the N-terminal kinase domain, leading to kinase activation. RhoA-independent mechanisms of activating the ROCKs have also been described ^3– 5^. Since ROCK1 and ROCK2 exhibit a high degree of identity in their kinase domains, they can phosphorylate similar substrates *in vitro* ^3– 5^. Isoform-specific roles of the ROCKs *in vivo* are, however, likely to exist and may become fully appreciated once detailed analyses of ROCK1 and ROCK2 conditional knockout mice are undertaken.

The ROCKs control a diverse range of biological processes enabling them to act as critical coordinators of a tissue response to stress and injury. The regulation of cytoskeletal dynamics is one of the best-described roles of the RhoA-ROCK pathway, with the ROCKs being implicated in the control of several processes including actomyosin contractility, intermediate filament assembly, microtubule dynamics, and the tethering of integral membrane proteins to the actin cytoskeleton ^3– 5^. Consistent with its role in regulating cytoskeletal dynamics, the RhoA-ROCK pathway is also involved in establishing front-rear polarity and cell migration ^3– 5, 7^. The ROCKs have, furthermore, been shown to control cell proliferation and survival, although this regulation appears to be highly cell type and context dependent ^3– 5^. The ROCKs also regulate gene expression by controlling the nuclear translocation of transcription factors via effects on actin dynamics as well as by directly phosphorylating transcriptional activators and coactivators to alter their activity ^3– 5, 8^. In the following sections, we will first provide a brief overview of the roles of RhoA and/or the ROCKs in the development and activation of T and B cells. We will then highlight new findings potentially linking RhoA and/or the ROCKs to autoimmunity and lymphoproliferative disorders.

## The RhoA–ROCK pathway in T and B cell development

Early studies employing a number of transgenic models demonstrated a role for RhoA in thymocyte development ^9, 10^. More recently, T-cell-specific conditional knockout mice generated by crossing RhoA ^flox/flox^ with *CD2-Cre* or *Lck-Cre* transgenic mice have confirmed these early findings and further shown that the absence of RhoA leads to defective thymocyte β-selection, impaired positive selection, and decreased thymocyte proliferation and survival ^11^. These abnormalities were accompanied by reduced pre- T cell receptor (TCR) expression, impaired TCR signaling, enhanced mitochondrial function, and increased ROS production, suggesting a multifaceted and complex role of RhoA in thymocyte development ^11^. The downstream effectors mediating the diverse effects of RhoA on thymocyte development have not been investigated and thus it is not yet known whether the ROCKs, or other effectors, are directly involved in mediating the RhoA-dependent T cell developmental actions.

RhoA plays a non-redundant role in B cell development, as underscored by the marked reduction in peripheral B cell populations (encompassing transitional, follicular, and marginal zone B cell compartments) that occurs when RhoA is deleted using a *CD19-Cre* transgene ^12^. The effects of RhoA deficiency on peripheral B cells were shown to be due to decreased expression of the BAFF receptor (BAFF-R) leading to defects in BAFF/BAFFR-mediated survival, while B cell receptor (BCR)-mediated survival was unaffected by the absence of RhoA ^12^. Although a detailed examination of the role of the ROCKs in B cell development has not been conducted, the addition of a ROCK inhibitor was shown to partially blunt the response of normal B cells to BAFF, suggesting that the effects of RhoA on BAFF-mediated B cell survival may partly rely on ROCK activation ^12^.

## The RhoA-ROCK pathway in T and B cell activation

In response to antigen exposure, the adaptive immune system undergoes a rapid and coordinated response geared at clearing the insulting pathogen. In addition to the expansion of antigen-specific T and B cells and the acquisition of specific differentiation states, these responses also rely on complex cytoskeletal rearrangements to regulate cell migration and cell-cell interactions. Not surprisingly, given the involvement of the RhoA-ROCK pathway in cytoskeletal reorganization, studies exploring the role of RhoA in T cell activation have primarily focused on its role in the regulation of cytoskeletal dynamics. In line with early studies showing that the RhoA-ROCK pathway is activated downstream of chemokine receptors such as CXCL12 ^13– 15^, the utilization of a RhoA activity biosensor has demonstrated that active RhoA can be detected at the leading edge in lamellipodia and filopodia, as well as in the uropod of migrating T cells ^16^. Importantly, as reviewed in more detail in 7, RhoA and its downstream effectors, including the ROCKs, are required for transendothelial migration (TEM) by promoting uropod contractility and by modulating integrin-mediated T cell adhesion. T cell transmigration through endothelial cells with low, but not high, permeability appears to be particularly reliant on the RhoA-ROCK pathway because of the greater dependency of T cell migration on effective T cell uropod contractility in the former settings ^17^. Involvement of the RhoA-ROCK pathway in additional T cell cytoskeletal processes such as the regulation of lipid raft dynamics has also been suggested ^18, 19^, although more work will need to be performed to fully address the role of the RhoA-ROCK pathway in these aspects of T cell biology.

Following early leads suggesting a role for the RhoA-ROCK pathway in the proliferation and activation of T cells ^19, 20^, recent studies have furthermore implicated the RhoA-ROCK pathway in the regulation of T helper (T _H_) cell differentiation. The lack of RhoA in T cells has been shown to impair T _H_2, but not T _H_1, differentiation *in vitro*, presumably by modulating metabolic processes such as glycolysis ^21^. These effects may be mediated by the ROCK1 isoform, since heterozygous ROCK1-deficient mice exhibit decreased expression of the T _H_2 cytokines interleukin (IL)-5 and IL-13 in bronchoalveolar lavage (BAL) fluid from a murine model of allergic inflammation ^22^. In contrast, ROCK2 is selectively activated under T _H_17-skewing conditions, but not under neutral, T _H_1, or T _H_2 conditions, and phosphorylates IRF4, a key regulator of IL-17 and IL-21 production ^23^. In line with these results, naïve T cells from heterozygous ROCK2-deficient mice exhibit impaired T _H_17 differentiation, as demonstrated by decreased expression of RORγt and diminished production of IL-17 and IL-21 ^23^. Findings in the murine system have been corroborated by human studies showing increased ROCK activation in human T cells exposed to T _H_17 conditions and a similar dependency of human IL-17 and IL-21 production on the ROCK2 rather than the ROCK1 isoform ^24, 25^. The addition of a selective ROCK2 inhibitor to differentiating human T cells can furthermore skew the T _H_17-Treg balance by decreasing the activation of STAT3 while promoting that of STAT5, suggesting that ROCK2 can control T _H_17 differentiation by multiple mechanisms ^25^. This modulation of STAT3 signaling may also underlie the recently reported ability of ROCK2 to regulate the *in vitro* differentiation of follicular T helper cells generated under T _H_17-skewing conditions ^26^. While additional studies will be required to fully define the precise role of the two ROCK isoforms in different T _H_ subsets, these initial investigations suggest that ROCK1 and ROCK2 may promote the differentiation of distinct T _H_ subsets.

Similarly to T cells, the most extensively characterized role of the RhoA-ROCK pathway in the B cell compartment lies in the regulation of cytoskeletal reorganization, although an involvement of RhoA in the regulation of BCR-induced proliferation of mature B cells has also been described ^27^. Studies examining BCR dynamics *in vitro* have shown that active RhoA interferes with the ability of TLR ligands to enhance BCR signaling by restricting BCR mobility via effects on the actin-severing protein cofilin ^28^, while ROCK1 activation is required for antigen internalization through the BCR ^29^, suggesting a dynamic utilization of this axis in response to distinct B cell stimulatory pathways. Not surprisingly, the RhoA-ROCK pathway has also been shown to regulate the migration of B cells ^30– 32^. Intriguingly, recent studies have uncovered a role for one of the Rho-GEFs, ARHGEF1, in the retention of B cells within the germinal center (GC) ^33^. Potentially relevant to the recently described involvement of RhoA in lymphomagenesis, which will be discussed below, the lack of ARHGEF1 in GC B cells was accompanied by the systemic dissemination of GC B cells out of the mesenteric lymph nodes ^33^. Whether the pro- or anti-migratory roles of RhoA in mature B cell populations or GC B cells are mediated by distinct RhoA effectors remains to be determined, but could be of great interest for the proper therapeutic targeting of this pathway.

## The RhoA–ROCK pathway in autoimmunity

While dysregulation of the RhoA-ROCK pathway has been well documented in cardiovascular, renal, and neurological disorders ^34– 36^, its impact on the pathogenesis of immune-mediated diseases is just beginning to be appreciated ^8^. In line with a broad role for T _H_17 cells in autoimmunity, and consistent with the ability of the RhoA-ROCK pathway to regulate this T _H _subset, aberrant activation of this pathway has been observed in murine models of rheumatoid arthritis (RA), systemic lupus erythematosus (SLE), and multiple sclerosis (MS). Indeed, T cells from a spontaneous mouse model of RA exhibited increased activation of ROCK2 and dysregulated production of IL-17 and IL-21, which was shown to be dependent on both RhoA and ROCK2 ^23^. Enhanced ROCK activation has also been observed in synovial tissues from rodents with collagen-induced arthritis, an induced model of arthritis ^37^. Notably, *in vivo* administration of a pan-ROCK inhibitor, fasudil (which blocks both ROCK1 and ROCK2 activity), or a ROCK2 selective inhibitor resulted in decreased IL-17 and IL-21 production, diminished autoantibody production, and attenuation of arthritis in both spontaneous and induced models of RA ^23, 25^, supporting a role for the RhoA–ROCK pathway, and in particular the ROCK2 isoform, in RA.

T cells from MRL/lpr mice, a spontaneous model of lupus, also display aberrant activation of ROCK2, and ROCK inhibition diminishes their *in vitro* production of IL-17 and IL-21 ^23^. Administration of the pan-ROCK inhibitor fasudil furthermore diminished the production of these cytokines *in vivo* and resulted in remarkable improvements in autoantibody production and proteinuria ^23^. The administration of fasudil to NZB/W F1 mice, a distinct spontaneous model of lupus, was accompanied by decreased plasma cell formation and also resulted in lower levels of autoantibodies and protection from nephritis ^38^. ROCK dysfunction may also contribute to the pathogenesis of MS. Increased ROCK activity has indeed been observed in the spleens and spinal cord of mice following the induction of EAE, a rodent model of MS, and the administration of ROCK inhibitors can delay disease onset and severity via a number of mechanisms including inhibition of IL-17 production ^39– 41^ and induction of regulatory T cells ^40, 41^.

Human studies also support the notion that dysregulated ROCK activation might contribute to autoimmunity. Enhanced phosphorylation levels of ROCK substrates, like the ERM proteins, have been observed in T cells from SLE patients ^42^. Furthermore, approximately 60% of SLE patients display higher levels of ROCK activity in their peripheral blood mononuclear cells (PBMCs) than do healthy controls ^24^. The production of IL-17 and IL-21 by SLE T cells is furthermore amenable to inhibition by statins (which, by blocking RhoA prenylation, can interfere with RhoA activation), a pan-ROCK inhibitor, or a selective ROCK2 inhibitor, further supporting a link between the RhoA–ROCK pathway and T cell dysfunction in this disease (Rozo, Salmon, and Pernis, unpublished observations). PBMCs from RA patients also display enhanced ROCK activity compared to healthy controls (Khianey Maharaj, Rozo, Bykerk, Goodman, and Pernis, unpublished observations), and a selective ROCK2 inhibitor similarly diminished IL-17 and IL-21 production by RA T cells ^25^. Studies in patients with relapsing remitting MS have furthermore shown that the production of T _H_17 cytokines by T cells from these patients could be inhibited by statins in addition to a pan-ROCK inhibitor ^43^. Dysregulation in the RhoA-ROCK pathway may thus represent a common pathogenic mechanism in multiple autoimmune disorders.

## The RhoA–ROCK pathway in lymphomagenesis

Signaling downstream of Rho-GTPases has been shown to play critical roles in regulating several aspects of tumorigenesis and metastasis including proliferation, survival, and invasion ^44, 45^. Consistent with its multifaceted role in regulating these key processes, aberrancies in the RhoA-ROCK signaling pathway have been observed in several non-hematopoietic and hematopoietic malignancies and have often been associated with the overexpression of Rho family members or activating mutations in the ROCKs ^44– 46^. Intriguingly, recent studies have demonstrated that inactivating mutations in RhoA can promote lymphomagenesis. Indeed, approximately 60–70% of angioimmunoblastic T-cell lymphoma cases, a rare peripheral T cell lymphoma that phenotypically resembles follicular T _H_ cells, have been found to express an inactivating mutation in RhoA (encoding p.Gly17Val) ^47– 50^. The Gly17Val RhoA mutant does not bind GTP and is believed to act as a dominant-negative by sequestering activated GEFs ^51^. Similar mutations in the GTP-binding domain of RhoA have also been observed in cutaneous T cell lymphoma (CTCL) ^52^. As additional investigations of this pathway in T cell lymphomas are being undertaken, a more complex situation is, however, emerging. Both loss- and gain-of-function RhoA mutations have recently been identified in adult T cell leukemia/lymphoma, which interestingly may be associated with distinct T cell phenotypes ^53, 54^.

Potentially inactivating mutations in components of the RhoA pathway, including RhoA itself and the RhoA-GEF ARHGEF1, have also been reported in two B cell lymphomas, Burkitt’s lymphoma (BL) and GC B-cell-derived diffuse large B cell lymphoma (DLBCL) ^33, 55, 56^. RhoA mutations in BL are commonly found within the GEF-binding domain and inhibit the ability of RhoA to bind to and become activated by GEFs ^56^. Interestingly, RhoA mutations are more prevalent in endemic BL compared to sporadic BL, and they overlap with those detected in peripheral T cell lymphomas ^57^. Given that both types of tumors are associated with Epstein-Barr virus (EBV) infection, it will be important to determine whether the cross-talk between RhoA-mediated pathways and EBV could help promote lymphomagenesis ^57^.

In line with the ability of the RhoA-ROCK pathway to regulate cytoskeletal dynamics, the migration of several B cell malignancies, including classic Hodgkin lymphoma (cHL), chronic lymphocytic leukemia (CLL), and multiple myeloma (MM), was shown to depend on ROCK signaling *in vitro* ^15, 30, 31, 58^, and homing of MM cells to the bone marrow in xenograft models could be prevented by pre-treatment of cells with a ROCK inhibitor ^15^. However, the activation of RhoA may not necessarily be linked to the dissemination of tumor cells but rather may suppress migration. Indeed, mice lacking Gα13, an upstream regulator of the RhoA-ROCK pathway and common target for mutations in BL ^33, 56, 59^, develop B-cell-derived lymphomas characterized by the dissemination of GC B cells from the lymph nodes into the periphery ^33^. Similarly, mutations in ARHGEF1, which mediates the activation of RhoA in response to Gα13 and other receptors, have also been identified in GC-derived B cell lymphomas and, as mentioned above, the absence of ARHGEF1 in mice also results in the dissemination of GC B cells from the mesenteric lymph nodes into the periphery ^33^. Likewise, GC-derived DLBCLs expressing high levels of HGAL, a protein that binds to and enhances the activity of RhoA-GEFs, exhibit high levels of RhoA activity, which can suppress cell migration *in vitro* ^60, 61^. The contrasting roles of RhoA activation, whereby it can suppress the migration of tumor cells from the lymph nodes in the case of BL yet promote homing to the bone marrow in the case of MM, underscore the complexity of RhoA biology and highlight the dynamic ability of RhoA-controlled pathways to be influenced in a cell-type-specific manner.

## Conclusions

The RhoA-ROCK pathway is a central coordinator of tissue injury response. Recent findings have shown that in addition to its well-known roles in regulating the non-hematopoietic compartment, the RhoA-ROCK pathway is also critical for the recruitment and function of immune cells, including T and B cells, to sites of tissue damage. The multifaceted involvement of the RhoA-ROCK pathway in T and B cell biology has resulted in an increasing appreciation that dysregulation of this pathway may play potential pathophysiological roles in autoimmune and lymphoproliferative disorders. While this commentary has focused on a select few conditions, recent work in scleroderma, vasculitis, and graft-versus-host disease ^62– 65^ suggest an extensive involvement of this pathway in a wide spectrum of immune-mediated diseases. Future studies coupling genetic approaches to the growing number of non-selective and selective pharmacologic agents that are becoming available to target this pathway will be invaluable to unravel the intricacies of the RhoA-ROCK pathway in different immune-mediated pathophysiological states. This information, in turn, will be essential to ensure that this pathway, which could be highly amenable to therapeutic intervention, is effectively targeted in autoimmune and lymphoproliferative disorders.
